# Supplementary material for: Are carbon emissions trading and green financial instruments synergistic? -Comprehensive quantitative research based on content analysis
Source: PLoS One. 2024 Mar 7;19(3):e0298601. doi: 10.1371/journal.pone.0298601 (PMC10919601; doi:10.1371/journal.pone.0298601)
Supplement: S1 Data — (ZIP) [file pone.0298601.s001.zip › Data/地级市Z_F工作报告.zip!/╔╜╢1⁄2/═■║ú╩╨/└┤╫╘╣1⁄2╓┌║┼í╛┬φ┐╦╩2╛▌═°í┐_2003.docx]

各位代表：

　　现在，我代表威海市人民政府向大会做工作报告，请予审议，并请各位政协委员和其他列席人员提出意见。

　　一、过去五年政府工作的基本回顾

　　过去的五年，是我市改革开放和现代化建设取得重大进展的五年。五年来，在省委、省政府和市委的正确领导下，本届政府团结和带领全市人民，以邓小平理论和“三个代表”重要思想为指导，认真贯彻党的十五大精神，解放思想，抢抓机遇，同心同德，锐意进取，圆满完成了市第十三届人大确定的各项工作任务，为新世纪的发展奠定了坚实的基础。

　　——国民经济持续增长，综合实力明显增强。五年间国内生产总值年均递增12.6%，其中第一产业递增4.8%，第二产业递增14.6%，第三产业递增14%。2002年全市国内生产总值达到703.5亿元，比上年增长14.5%，是1997年的1.8倍，人均达到2.8万元，按目前汇率约合3400美元。

　　——结构调整步伐加快，经济运行质量逐年提高。三次产业比例由1997年的24：47：29调整为2002年的13：55：32。农业结构调整取得较大突破，粮经面积比由72：28调整到44：56，渔业养殖与捕捞产量比由48：52调整到54：46，森林覆盖率平均每年增加1个百分点，2002年达到32.7%。工业结构调整全面推进，机械、化工、建材等传统产业普遍得到改造，电子信息、新材料、生物工程等高新技术产业快速成长，骨干优势企业群体不断壮大，运行质量和效益进一步提高。县以上单位五年累计完成技改投资175亿元，其中2002年完成40.3亿元，比上年增长92.7%。高新技术产业产值占工业总产值的比重达到14.7%；销售收入过亿元、利税过千万元的企业达到276家和268家，五年分别增加186家和157家；限额以上工业产品销售收入年均递增19.4%，2002年突破千亿元大关，达到1026亿元；实现利税年均递增16.5%，2002年达到104.7亿元。三产内部结构得到优化，现代物流、现代旅游等新兴服务业发展速度明显加快，社会消费品零售总额年均递增10%，2002年达到188.6亿元，新型流通业态和现代营销方式所占比重达到19.7%。旅游收入年均递增19%，2002年达到55.5亿元。

　　——对外开放向纵深推进，成为经济增长的主要动力。加强了两个国家级开发区及各类园区建设，设立了威海出口加工区、高技术产品出口基地，新开通了三条直达韩国的海上客货班轮航线，成功举办了中韩经贸洽谈会、东北亚经济论坛等一系列重大涉外活动，改进了招商方式，拓宽了招商领域，突出了重点市场、重点企业和重点产品出口，外经外贸再创新高。2002年，引进投资过千万美元的外资项目26个，比上年增加15个。全年合同利用外资11.4亿美元，比上年增长115.8%；实际利用外资6亿美元，增长76.9%。五年累计合同利用外资25.2亿美元，相当于前十年的总和；累计实际利用外资15.1亿美元，是前十年的1.3倍。外贸进出口总额年均递增12.8%，2002年达到29.3亿美元，比上年增长23.2%，其中出口19.2亿美元，增长20.6%。五年来共完成对外承包劳务营业额2.7亿美元，外派劳务1.4万人次，期末在外人数6100多人。

　　——各项改革配套推进，社会主义市场经济体制初步建立。企业产权制度改革取得重大突破，多种形式的股份制改造迈出较大步伐，企业改制面达到81.4%。公司上市有新的进展，虎山粮机在主板吸收合并上市，华新海大在香港创业板上市已获中国证监会批准，医用高分子等5家公司上市申报材料已被正式受理。财税、流通、物价、住房、政府机构等项改革取得新的进展。制定了鼓励个体私营经济发展的政策措施，民营经济呈现出旺盛的发展势头，从业人员和注册资本五年分别增长了1.1倍和4.2倍，达到17.2万人和58.6亿元。进一步完善了社会保障制度，“两个确保”得到巩固，“三条保障线”制度逐步完善，市场导向的就业机制基本形成，城镇职工登记失业率始终控制在1.7%以内。农村第二轮土地承包顺利进行，农村税费改革全面推行，农民人均减负46元。

　　——基础设施和城乡建设步伐加快，发展环境不断优化。五年共完成全社会固定资产投资566亿元，是前五年的1.7倍，其中2002年完成187.2亿元，比上年增长63.9%。新上了1000多个投资过千万元的大项目，建成了309国道威海段一级路、威海电厂二期、威海港新区二期、米威调水二期、城乡电网改造等30多项重点工程，经济发展的支撑条件进一步改善。以加快城市化步伐为目标，调整了城镇布局，实施了城市道路建设、环境绿化美化、垃圾污水处理、公用设施建设等工程，初步形成了“中心城市—次中心城市—中心镇”三个层次的城镇体系，建制镇由1997年的72个调减到52个，城市化率由38.7%提高到49.7%。中心城市规模不断扩大，建成区面积达到43.7平方公里，集聚和辐射能力不断增强。围绕服务环境的优化，设立了行风热线，推进了行政审批制度改革，建立了市级行政审批中心，实施了政府“提速”工程，开展了“投资环境优化年”、“优政建设年”等活动，机关作风有了新的转变，服务质量和行政效率有了较大提高。

　　——财政收入大幅度增长，金融形势逐步好转。按可比口径计算，五年间地方财政收入年均递增21.9%，2002年达到28.6亿元，比上年增长22.7%。积极推进政府采购制度、公费个人支出方式等项改革，提高了财政资金的使用效益，较好地保证了重点支出需要，连续16年实现了收支平衡。以创建金融安全区为抓手，不断改进信贷方式，努力提高资产质量和服务水平，成功摘掉了金融高风险区的帽子。到2002年底，全市本外币贷款余额达到342.7亿元，比上年增长19.1%；本外币存款余额达到467.8亿元，增长15.1%。

　　——社会事业迈出新的步伐，精神文明建设取得可喜成绩。组织实施市级以上科技计划1031项，取得重大科技成果263项，解决了一批制约经济发展的技术“瓶颈”，被评为“全国科技工作先进市”，被确定为“全国信息化试点城市”。高等教育取得较快发展，与山大威海分校、哈工大威海校区共建工作成效显著，威海职业学院、技术学院建设初见成效。基础教育进一步巩固，素质教育全面推进，学前教育、九年制义务教育和高中阶段教育入学率分别达到85.7%、99.7%和89.9%，普通高考本科上线率和人口万人比连续9年居全省首位。多方筹集社会救济、福利资金，不同程度地解决了困难群众和优抚对象的生产、生活问题。建立了土地“五统一”管理制度，强化了环境保护，人口自然增长率连续四年保持负增长。加强了“双拥”和民兵预备役工作。依法治市、依法行政进一步推进，民主政治建设得到加强，村民自治制度在农村普遍推行。深入开展反腐败斗争，查办了一批违法违纪案件。坚持“打防并举、标本兼治”方针，社会治安综合治理取得明显成效。大力开展了精神文明创建活动，创建文明城市、文明行业、文明村镇工作跨入全国先进行列，三市一区连续四届被评为“全省精神文明建设先进县（市、区）”。文化、卫生、民政、广播电视、新闻出版、民族、宗教、外事、侨务、档案、史志、气象、减灾、老龄、残疾人等事业也都有了新的发展。

　　——城乡居民收入稳步增加，人民生活水平显著提高。五年间全市城镇居民人均可支配收入和农民人均纯收入年均分别递增15.4%和6.2%，2002年分别达到9390元和4402元。城镇和农村居民人均住房面积分别由1997年的11.5平方米和25平方米，增加到14.7平方米和27平方米。固定电话用户发展到77万户，移动电话用户发展到48.8万户，互联网用户发展到11万户。居民消费结构发生较大变化，用于教育、文化、保健和旅游等方面的支出逐年增加，恩格尔系数由31.9%下降到29%。

　　各位代表，世纪之交的五年，是威海发展史上很不平凡的五年。这五年，改革处在攻坚阶段，发展处在关键时期，情况新而复杂，困难多而集中，我们先后遇到了亚洲金融危机、连续三年特大干旱、金融风险、市场疲软等多种困难。在严峻的形势面前，全市上下团结一心，群策群力，在攻坚破难中实现了经济社会的大发展，在奋勇争先中赢得了新的荣誉。五年来，荣成、文登、乳山全部跻身于全国百强县行列，我市的经济总量和人均值在全省继续保持先进位置，并先后被授予“全国创建文明城市工作先进单位”、“全国环境综合整治优秀城市”、“中国优秀旅游城市”等称号，获得了首届“中国人居环境范例奖”和“山东人居环境奖”，成为全国第一个国家环境保护模范城市群，并再次被联合国评为“迪拜国际改善居住环境最佳范例”城市。五年的辉煌令人振奋，五年的成就来之不易。回首过去五年的工作，我们深切感到，党中央、国务院和省委、省政府正确的方针政策，市委的坚强领导，人民群众的信任支持，全市上下的团结奋斗，是我们不断战胜困难、经受考验、夺取胜利的根本保证。在此,我代表市人民政府，向在各条战线上辛勤劳动、作出贡献的全市人民，向给予我们大力支持与监督的人大代表、政协委员、各民主党派和无党派人士、各人民团体、社会各界人士，向大力支持我们工作的驻威部队、武警官兵、中直省直驻威单位，向所有关心支持威海建设的港澳台同胞、海外侨胞、国内外各界友人表示崇高的敬意和衷心的感谢！

　　各位代表，五年的实践，进一步深化了我们对威海现代化建设的规律性认识，积累了许多宝贵经验。第一，必须坚持用邓小平理论和“三个代表”重要思想武装干部群众、指导工作实践，把解放思想、与时俱进、开拓创新，作为推进事业发展的根本动力。第二，必须始终坚持以经济建设为中心，聚精会神搞建设，一心一意谋发展，遵循经济发展的客观规律，创造性地开展工作，努力走出一条具有时代特征和威海特色的发展路子。第三，必须认清比较优势，把握关键环节，不断深化改革，突出对外开放，抓好科技和人才，全面优化发展环境，努力增创新优势，实现发展的新跨越。第四，必须正确处理好改革、发展、稳定的关系，把改革的力度、发展的速度和社会可承受的程度有机统一起来，在社会稳定中推进改革与发展，在改革与发展中保持社会稳定。第五，必须充分调动、保护和发挥方方面面的积极性，尊重群众的首创精神，集中精力抓落实，心情舒畅干工作，营造干事创业的良好社会氛围。第六，必须把为人民谋利益作为一切工作的出发点和归宿，心系百姓，执政为民，切实维护最广大人民的根本利益。第七，必须坚持经济社会整体推进、协调发展，处理好经济发展与人口、资源、环境建设的关系，实现物质文明与精神文明、经济社会与生态环境的和谐与可持续发展。

　　各位代表，总结过去的五年，我们同时也清醒地看到发展中存在的矛盾和问题。主要是：经济规模和总量还不大，经济结构还不够优化；科技创新能力还不强；改革还没有完全到位，发展的体制机制性障碍仍然存在；开放的水平还不够高，投资环境还需进一步改善；部分群众的生产和生活还比较困难，企业脱困、社会保障、扩大就业、农民增收、城镇建设等方面的任务仍很艰巨；政府职能转变还不到位，少数部门和同志思想还不够解放，服务意识不强，存在不正之风和腐败现象，等等。对此，我们必须高度重视，并在今后的工作中认真加以解决。

　　二、新一届政府的工作目标和任务

　　各位代表，党的十六大确立了全面建设小康社会的奋斗目标，并指出有条件的地方可以发展得更快一些，率先基本实现现代化，这为我们今后的工作指明了前进的方向。刚刚闭幕的市十二届一次党代会，确定了我市本世纪头二十年的奋斗目标：到2020年国内生产总值在2000年的基础上翻三番。第一步，到2005年翻一番，超过1000亿元，人均4000美元以上，基本实现建设全面小康社会的奋斗目标；第二步，到2012年翻两番，超过2000亿元，人均8000美元以上，基本实现社会主义现代化；第三步，到2020年翻三番，超过4000亿元，人均15000美元以上，达到世界中等发达国家发展水平，建设成为经济繁荣发达、科技教育先进、生态环境优良、民主法制健全、社会文明进步、人民生活更加富裕的新威海。

　　实现“翻三番”，是威海本世纪头二十年的总目标，新一届政府担负着建设全面小康社会，为基本实现现代化开好头、起好步、夯实基础的艰巨任务。总的工作要求是：以邓小平理论和“三个代表”重要思想为指导，全面贯彻党的十六大精神，认真落实市十二届一次党代会提出的各项任务，坚持把发展作为第一要务，大力实施经济国际化、城市化、“科教兴威”和可持续发展战略，更加有效地抓好对外开放，以开放促观念更新、促体制改革、促结构调整、促科技进步、促资金投入、促各项事业的全面发展，为实现“翻三番”的宏伟目标而努力奋斗。

　　主要任务是：

　　——实现国民经济跳跃式发展。国内生产总值年均增长14%以上，全社会固定资产投资年均增长16%以上，地方财政收入年均增长16%以上。主要经济指标人均占有保持全省领先水平，经济总量在全省的位次力争前移。

　　——构建高素质的现代产业体系。三次产业的比重调整到10：54：36，形成以高新技术产业为先导、现代农业为基础、制造业为支撑、服务业全面发展的新格局，产业素质和国际竞争力显著提升，逐步使威海成为韩、日等周边国家和地区的工业品加工、制造和出口基地，农副产品供给基地，旅游休闲娱乐基地。

　　——建立比较完善的市场经济体制。充分发挥市场在资源配置中的基础性作用，健全更加统一、开放、竞争、有序的现代市场体系。各种所有制经济在市场经济中发挥各自优势，相互促进，共同发展，基本经济制度更加完善。宏观调控、社会保障、收入分配体系进一步健全。

　　——形成更高层次的开放格局。实际利用外资年均增长20%以上，外贸出口年均增长20%以上。国际吸引力、影响力、竞争力日益增强，使更具实力、更有活力、更加开放的威海全方位走向世界、融入世界。

　　——营造更具吸引力的发展环境。基础设施更加完善配套，生态环境和生活环境进一步改善，政府职能明显转变，规范的市场运行机制、良好的社会诚信体系和高效能的行政服务体系基本建立，“最适宜人类居住和创业”的品牌进一步打响。

　　——建设特色鲜明的文化城市。文化事业更加繁荣，文化产业长足发展，科技教育明显进步，公民素质进一步提高，各项事业全面发展，形成开放性与特色性和谐统一、现代文化与传统文化相互融合、大众文化与精品文化共同繁荣的威海文化。

　　——不断发展社会主义民主政治。社会主义民主进一步完善，社会主义法制比较完备，依法治市进一步落实，人民的法制意识普遍增强，政治、经济和文化权益得到切实尊重和保障，基层民主更加健全，经济和社会管理走上法制化、规范化的轨道，社会稳定，政治安定，人民安居乐业。

　　——建设更加富足的小康生活。城市化水平达到55%，城镇居民人均可支配收入年均增长8%，农民人均纯收入年均增长6%。基本实现充分就业，切实保障弱势群体的基本生活。城镇和农村的恩格尔系数分别降到25%和36%以下，人均期望寿命达到75岁以上。

　　实现五年的奋斗目标，关键是第一年。今年全市主要预期目标安排为：国内生产总值增长14%以上，限额以上工业增加值增长16%以上，地方财政收入增长15%以上，全社会固定资产投资增长25%以上，外贸出口增长20%以上，实际利用外资增长30%以上，社会消费品零售总额增长12%以上，城镇居民人均可支配收入增长8%以上，农民人均纯收入增长6%以上。我们应当看到，现在全国上下区域竞争日益激烈，呈现出百舸争流、千帆竞发、万马争先的发展态势，尤其是南方发达地区和省内周边市区，气魄很大，步子很快，上得很猛。在这种咄咄逼人的形势下，比发展速度的快与慢，慢就是退；比经济总量的大与小，小就是弱；比总体素质的高与低，低就是败。我们威海要争创新优势，赢得新发展，提高在全省、全国的经济地位，必须立足于大发展、快发展，积极有为，奋力争先，在着力提高经济运行质量和效益的基础上，能争取的速度一定要积极争取，能加快的步伐一定要千方百计加快，上述预期性、指导性指标能多超的一定要努力多超，真正实现我市经济的超常规、跳跃式发展。

　　各位代表，加快经济社会发展，建设全面小康和现代化的新威海，是党的重托、时代的呼唤、人民的期望。能在这千百年来人们梦寐以求的伟大事业中建功立业、造福人民，是我们难得的机遇，也是我们的光荣和自豪。所有政府工作人员都要倍加珍惜这一机遇，恪尽职守，奋发有为，惟加快是重，惟加快是图，敢与强的比，敢向高的攀，敢同勇的争，敢跟快的赛，以发展论英雄，以发展辨是非，以发展评得失，为加快威海的现代化建设，奉献出我们全部的忠诚、全部的心血、全部的汗水！我们相信，有省委、省政府和市委的坚强领导，有历届政府奠定的坚实基础，有全市人民的大力支持，我们的奋斗目标一定能够实现，威海的明天一定会更加美好！

　　三、今后五年和2003年政府工作重点

　　今后五年，是在新的历史起点上，建设全面小康社会、实现现代化建设新突破的关键时期，一定要按照“发展要有新思路，改革要有新突破，开放要有新局面，各项工作要有新举措”的要求，把对外开放作为龙头，把结构调整作为主线，把深化改革作为动力，把发展环境作为基础，把科技和人才作为条件，把提高人民生活水平作为目的，奋力推动经济和社会的全面、协调发展。

　　（一）积极扩大投资规模。投资是经济增长的动力和源泉，必须以招商引资为突破口，多渠道筹措发展资金，始终保持较高的投资强度。

　　一是抓住世界范围产业结构调整，发达国家制造业转移，尤其是韩国、日本制造业大举向中国转移的战略机遇期，更大规模、更高质量地引进外资。坚持主攻韩日、扩大港台、开拓欧美，全方位拓宽引资渠道。进一步优化外商投资结构，促其更多地投向高新技术产业、基础设施、农业等领域，加快服务领域的开放步伐。在扩大直接利用外资的同时，继续合理有效地利用跨国并购、股权转让、境外上市等多种方式，实现利用外资方式的新突破。加强招商网络建设，有选择地在海外设立招商联系点，发展招商经纪人队伍，实行委托招商。在继续抓好全社会招商的同时，面向世界500强企业，突出大项目招商，对投资过千万美元的项目，组建领导、部门、专家参与的推进小组靠上去抓。加快两个国家级开发区、出口加工区及其他各类园区建设，搞好功能创新，真正发挥示范、辐射和带动作用。五年内实际利用外资达到50亿美元以上。今年要突出抓好韩国招商月和环黄海经济研讨会等大型招商活动，重点搞好欧星显示器等项目建设，争取比利时钢丝帘线、天然气一期等大项目招商成功，全年引进过千万美元的外资项目40个以上，实际利用外资力争超过8亿美元。

　　二是广泛吸引国内资金。坚持威海以外都是外，内资外资一齐引，面向国有大企业、上市公司、民营企业及各类行业协会，加大国内知名企业、知名品牌的引进力度，五年内，实际利用内资年均递增13%以上，累计达到430亿元以上。今年，在搞好魏桥纺织二期、第一污水处理厂搬迁改造等大项目建设的同时，搞好与深圳高新技术产业投资公司等的合作，引进内资80亿元以上。

　　三是积极启动民间投资。进一步放宽市场准入，允许外资进入的领域，都要鼓励民间资本参与竞争。对有收费机制或投资收益比较稳定的基础设施和社会事业项目，积极吸纳民营资本进入。引导民营资本参与公有企业改组、改造和高新技术产业发展，参股或控股公有企业。同时，要抓住国家继续扩大内需的有利机遇，论证、培育和储备一批高层次、高水平的大项目，努力争取更多的国债资金和上级各种资金支持。

　　在扩大投资规模的同时，强化产业政策导向，把有限的资金用在先进生产力的发展上，用在产业结构的优化升级上，用在克服影响经济和社会发展全局的瓶颈制约上，坚决制止低水平的重复建设，坚决制止无效益的“形象工程”、“政绩工程”，坚决制止用财政资金搞楼堂馆所。

　　（二）全方位开拓国际国内市场。市场的空间就是发展的空间，必须进一步强化市场观念，充分利用好国际国内两种资源、两个市场，推动经济的快速发展。

　　一是大力开拓国际市场。优化出口产品结构，加快产品国际认证，提高农副产品、纺织品等传统骨干产品的出口档次和质量，提高机电产品、高新技术产品的出口比重。继续实施“市场多元化”战略，进一步巩固韩国、日本等传统市场，努力扩大欧美等高价位市场，大力开拓俄罗斯、东欧、南美、南非等新兴市场，推动我市产品直接进入跨国公司全球采购和连锁营销网络。积极推进外贸主体多元化，力争使有外贸经营权企业每年增加100家以上。同时要进一步搞好WTO等的培训工作，引导企业按照WTO规则参与国际竞争。到2007年，全市外贸出口规模达到45亿美元以上。利用国家降低关税的有利时机，大力引进国外先进技术、关键设备和原材物料，形成大进大出的外贸格局。依托城边、海边、路边和各类园区，发动市、县、镇、村一齐上，大搞加工贸易，力争加工贸易出口占出口总额的比重每年提高2个百分点，到2007年达到70%左右。今年重点组织企业参加十大世界性博览会，扩大高附加值产品出口，力争机电产品出口增长45%以上，占出口总额的比重达到30%以上；高新技术产品出口增长50%以上，比重达到18%以上。

　　二是大力开拓国内市场。国内市场潜力巨大，是世界经济竞争的热点，决不能轻视，更不能忽视。要引导企业加强营销体系建设，紧紧依靠科技进步，实施品牌战略，以质量创品牌，以品牌拓市场，以市场兴产业。五年内争创国际名牌2个以上，中国名牌或驰名商标5个以上。要充分发挥政府和各经济部门的组织协调作用，把各类企业动员起来，形成整体优势，有计划、有针对性地搞好国内市场开拓。引导企业与超级市场、连锁企业建立直接供货关系，今年要组织好省里筹办的五大展销会，提高国内市场占有率。

　　三是大力开拓劳务市场。积极实施“走出去”战略，鼓励和支持有条件的企业，特别是木工机械、渔竿、水产养殖等优势行业的企业，到境外兴办加工贸易和资源开发项目，带动技术、设备、劳务输出。在巩固好韩国、日本等传统劳务市场的同时，重点开拓西欧、中东等劳务市场，今年对外承包劳务营业额增长20%以上，外派劳务增长20%以上。积极创造条件，组织、承办、参加国际性经济、技术、学术和文化等重大活动，扩大对外交流，提高国际化程度。在鼓励走出国门的同时，引导施工企业走向市外，到国内一切有商机的地方承包工程，求得发展。

　　（三）进一步深化市场取向的改革。以产权制度改革为核心，采取灵活多样的形式，搞好困难企业、镇村企业、基础设施、公益性行业的改革，力争五年内全部完成企业改制任务，其中今年企业改制面达到85%以上。按照《公司法》的要求，抓好改制企业的规范运作。加快大中型企业股份制改造，搞好公司上市工作，力争五年内上市企业达到10家以上，今年重点抓好6户企业的上市发行审核和申报工作，抓好3户股份有限公司的上市辅导工作。

　　按照党的十六大精神和上级的统一部署，结合我市实际，深化公有资产管理体制改革，按照管资产和管人、管事相结合的原则，健全公有资产管理和运营机构，理顺公有资产管理委员会、公有资产经营公司和参股控股企业的关系，完善绩效考核奖惩激励机制，实行授权经营，提高运营效率，确保公有资产保值增值。

　　强化市场在资源配置中的基础性作用。加快发展资本、产权、土地、劳动力和技术等生产要素市场和各类中介组织，建立和维护统一开放、竞争有序的市场体系。建立价格决策听证制度、价格审批和价格行政处罚集体审议制度。坚持一次分配讲效率、二次分配讲公平，完善资本、技术和管理等生产要素参与分配的办法，扩大中等收入者比重，提高低收入者收入水平，促进共同富裕。继续深化流通、财税、金融体制改革，积极稳妥地搞好文化、教育、卫生等社会事业的改革。

　　（四）加大产业结构调整力度。提高经济运行的质量和效益，产业结构调整是关键。要紧紧抓住国内外市场需求的新变化，积极推进产业结构的战略性调整，以质的提高赢得发展的主动权。

　　一要加快工业结构优化升级。确立工业在产业结构调整中的主导地位，坚持以信息化带动工业化，以工业化推进农业产业化、流通现代化，走新型工业化道路，形成优势产业加速成长的新格局。以制造业为基础，以技术改造为突破口，依托优势企业，加快对机械、化工、轻工、建材、纺织服装等传统行业的嫁接改造，培育汽车零部件、船舶修造、农副产品加工、医药等特色产业，形成新的产业集群和区域性制造加工中心。积极实施大公司、大集团战略，加大对34家省、市级重点骨干企业的扶持力度，引导企业资产合理流动和重组，培育出销售收入过10亿元的企业集团20家以上，过50亿元的5家以上，力争培育出过百亿元的大型企业集团。今年重点组织实施好9个国债技改项目，集中力量抓好三角集团130万套全钢子午胎等投资额过5亿元的技改项目，促其尽快投产见效，全年完成技改投资85亿元以上。

　　二要大力发展现代农业。坚持以市场为导向，以增加农民收入为目的，把农业产业化经营、农业标准化生产、小城镇建设有机结合起来，加快推进农业和农村经济结构的战略性调整。突出品种、品质和品牌，大力发展特色林果业、蔬菜业和畜牧业，形成区域优势。坚持绿化与美化相结合，大力实施“五林”工程，搞好40万亩速生杨基地和绿色通道建设，确保“十五”期间每年植树2000万棵，森林覆盖率每年提高2个百分点，2007年达到40%以上。加大对农业龙头企业的扶持力度，进一步加快农业产业化步伐，今年争取再有1-2家列入全国重点农业龙头企业行列，5家以上进入省级重点农业龙头企业行列。加强农业基础设施建设，不断改善农业生产条件，今年新发展节水灌溉面积10万亩。搞好小城镇规划建设，引导农村剩余劳动力向非农产业转移，向小城镇聚集，加快城乡一体化步伐。

　　三要大力发展海洋经济。积极实施“海上威海”战略，鼓励不同层次、不同所有制企业共同参与海洋资源开发。调整捕捞结构，大力发展远洋捕捞，尽快形成生产、加工、运销一条龙的远洋渔业体系，今年新增远洋渔船30艘以上。以名优品种为主，不断扩大海水养殖，大力发展海洋化工、食品、保健品等海洋加工业，今年新增深水网箱50个以上，建设一批高标准加工车间，名优养殖和精深加工比重分别达到45%和50%以上。加强威海和石岛两个水产品交易市场建设，巩固威海渔业主产区和主销区的地位。以全国海洋水产科技示范园、全国“科技兴海”示范基地为依托，加大投入，搞好新品种、新技术的引进、中试、孵化和推广工作，提升海洋经济的整体素质，实现由渔业大市向海洋经济强市的跨越，海洋经济增加值年均递增15%以上。

　　四要不断提高第三产业比重。坚持把旅游业作为龙头来抓，高起点制定并实施旅游业发展总体规划，开发建设一批具有国际水平的新、奇、特旅游观光项目。今年完成全市旅游发展总体规划，启动各市区旅游业发展规划，整合沿海岸线旅游资源，搞好刘公岛海权文化主题公园、世界渔村、温泉疗养等项目的论证与招商，开发渔家乐、海上之夜等旅游产品，推出四季游精品线路，争创全国第一个优秀旅游城市群，旅游业总收入增长17%以上。广泛应用现代营销手段，加快商贸流通、交通运输、宾馆餐饮等传统行业的改造。依托立体交通体系和独特区位优势，发展现代物流业，大力培育配送中心、连锁经营、仓储式超市等新的营销方式。积极发展信息服务、中介服务、社区服务、金融保险服务等新兴产业，提高服务业的整体档次和水平。今年在市区改造一处大型购物中心，规划建设一条商业步行街。

　　（五）加快高新技术产业发展。高新技术产业的发展，决定着威海经济发展的后劲和水平，要作为经济工作的一大亮点来抓。要立足威海的城市定位和产业优势，加快电子信息、生物技术、新材料、海洋技术四大新兴产业发展，尽快形成具有威海特色的高新技术产业体系。以北洋、三星、宏安集团等骨干企业为依托，大力发展微电子类产品、计算机配套类产品和网络通信类产品。以光威渔具、医用高分子和万丰奥威集团为依托，搞好碳纤维原丝、二氧化碳聚合物合成材料、高强镁合金等新材料的研制与生产，争创全省高分子和非金属材料生产基地。以国家海洋“863”计划产业化基地和清华紫光园为依托，力争在生物医药、海洋科技等领域有新的突破。到2007年，全市高新技术产业产值占工业总产值的比重达到20%以上。

　　增强自主创新能力。突出企业的技术创新主体地位，引导重点企业集团建立市级以上技术开发中心，大中型企业建立市级技术开发机构。今年重点抓好29家市级以上企业技术开发中心的规范和完善，组织好6家企业省级工程技术研究中心的申报工作，搞好三角轮胎等7家企业博士后工作站建设。

　　搞好产学研联合和人才开发。鼓励、支持企业加强与高等院校、科研院所和海外留学人员的联合，走产学研一体化的道路，逐步把威海建成全国重要的科技成果中试基地和产业化基地。今年重点扶持好黑豹集团电动车等一批重大产学研联合创新项目。进一步强化人才是第一资源的观念，尽快把发展的立足点由物质资源、人力资源转到科技资源和人才资源上来，建立和完善富有活力的人才聚集机制。不仅要注重引进人才，更要注重使用人才、留住人才，发挥人才的积极性和创造性。要分层次、多形式培养各类人才，特别是中青年人才，大力培养技能型人才，吸引国内外各类优秀人才以多种形式参与我市现代化建设，做到以人才兴科技，以科技兴产业，以产业求发展。

　　（六）大力发展民营经济。民营经济具有充足的活力、巨大的潜力和广阔的发展空间，是经济增长的动力所在。要按照党的十六大要求，进一步解放思想，更新观念，毫不动摇、理直气壮地鼓励、支持和引导非公有制经济发展，充分发挥民营经济在促进经济增长、扩大就业和活跃市场等方面的重要作用。

　　优化民营经济发展环境。彻底清除各类歧视性规定，放开手脚，降低门槛，取消限制，切实把国民待遇落到实处，凡是国家法律法规没有明令禁止的，都要允许民营企业生产经营；凡是国有、集体企业享受的待遇，民营企业同样享受。正确处理发展与规范的关系，严肃查处侵犯民营企业合法权益的行为。

　　加快膨胀民营经济规模。立足威海区域优势和产业优势，大力培植特色产业，五年内形成渔具、机械等几个百亿元的产业群。加大对现有骨干民营企业的培植力度，促其规模膨胀，五年内培植销售收入过亿元的民营企业100家以上，过10亿元的10家以上。依托市场、龙头企业和“三边”，建设和培育100个左右民营经济园区，实现民营经济集约化发展。鼓励民营企业到境内外上市融资。加强民营企业家队伍建设，推进民营企业体制创新、机制创新、技术创新，提高市场竞争能力。强化政策导向，鼓励公务员、企业管理人员和技术人员、下岗职工从事民营经济，力争5年内，全市个体户和私营企业发展到8.5万户以上，提供的税收占财政总收入的比重达到50%以上。

　　（七）搞好基础设施和城市建设。坚持高起点规划、高标准建设、高效能管理、高水平经营，完善城市功能，提高城市承载能力，增强城市综合竞争力。

　　优化城乡总体布局。把千公里海岸线、百公里旅游线和产业经济带布局纳入城市总体规划，增强规划的科学性、前瞻性、严肃性和权威性，规划一经批准，任何单位和个人都必须严格遵守，不得擅自更改。今年完成《城镇体系规划》、《城市总体规划》和《城市绿地系统规划》的修订、调整和报批工作，编制海岸线控制性详细规划，完成荣成、文登、乳山和12个中心镇总体规划的调整和报批工作，申报建设环海和里口山省级风景名胜区。

　　加快城乡基础设施建设。搞好桃威铁路扩能改造、威海港新区三期、威青高速公路、环海路、胶东调水和米威调水三期以及城镇污水处理厂扩建改造等工程建设，启动城市电子地理信息系统、教育局域网、互联网门户和电子政务建设。今年重点搞好八河水库续建、陆岛交通码头等14项重点工程建设，开工建设威海至乳山高速公路，全面启动村村通油路工程，完成解放路至烟台路二期改造、环山路绿化、体育基地迁建等工程。

　　提高城市经营水平。把城市作为最大的资产来经营，对城市土地、市政设施、无形资产等实行市场化运作，推进城市资产由公共资产向经营性资产转变。突出抓好土地使用制度改革，建立土地公开招标拍卖和挂牌出让等机制，整顿土地市场，管好土地资源，盘活土地资产，实现政府对土地一级市场的高度垄断。

　　搞好城市环境综合整治。始终坚持生态化方向不动摇，继续实施好“蓝天行动”、“碧海行动”、“绿色行动”，坚决保护好威海的青山绿水、碧海蓝天。继续搞好户外广告规范、街名路牌设置、建筑物清洗粉刷修缮及“平改坡”、“换新装”和“光亮工程”，健全城市无障碍设施，加快旧村改造和生活小区综合治理，巩固卫生城、环保模范城、园林城、人居范例城创建成果，争创“全球环境500佳”、“全国生态示范城市”和“联合国人居奖”。

　　（八）提高财政金融工作水平。抓好增收节支，把经济发展的成果切实体现在财政收入上，不断提高财政收入占GDP的比重。大力加强财源建设。加强对重点税源的监控和征收，强化税收征管，打击各类偷逃税行为。扩大预算管理范围，强化对土地收入、国有资产分红等非税收入的征管，集中财力办大事。牢固树立过紧日子的思想，调整优化支出结构，严格控制一般性支出，提高财政资金使用效益。

　　加强“诚信威海”建设，建立企业、经营者及个人信用体系，增强全社会的信用意识。深入推进金融安全区建设，严厉打击各种逃废银行债务行为，维护金融债权，进一步优化金融资产质量，确保金融和经济安全。认真贯彻稳健的货币政策，扩大信贷投放，力争各类贷款逐年有较大增长，支持全市经济发展。

　　（九）积极做好就业和社会保障工作。就业是民生之本。要把改善创业环境和增加就业岗位作为政府的重要职责，建立和完善促进就业的政府责任体系、政策法规体系、管理服务体系。立足在发展中解决就业问题，健全市场导向的就业机制，广开就业门路，落实各项促进就业的优惠政策，加快建立基层劳动保障工作平台，广泛开展再就业援助行动，千方百计帮助下岗失业人员实现再就业。搞好职业培训、上岗培训，提高劳动者就业能力和职业转换能力。今年，实现城镇就业4万人，转移农村剩余劳动力1.5万人，城镇登记失业率控制在2%以内。

　　进一步扩大养老、医疗、失业等社会保险覆盖面，逐步把非公有制经济从业人员纳入社会保险范围。坚持社会统筹和个人帐户相结合，多渠道筹集和积累社会保障基金。全面落实好“两个确保”和“一个低保”政策，努力做到应保尽保。积极发展城乡社会救济和社会福利事业，关心和帮助弱势群体、困难群众，千方百计为他们排忧解难。今年，城镇职工社会保险覆盖面达到90%以上，农村社会养老保险覆盖面达到60%以上。

　　（十）加强精神文明和民主法制建设，繁荣发展各项社会事业。坚持以德治市与依法治市相结合，健全社会主义民主与法制，推动社会主义物质文明、政治文明、精神文明协调发展。

　　加强社会主义思想道德建设。用邓小平理论和“三个代表”重要思想武装广大干部群众，认真落实《公民道德建设实施纲要》，大力宣传爱国守法、团结友善、勤俭自强、敬业奉献的道德规范，倡导社会公德、家庭美德和职业道德，培养和造就“四有公民”。加强科普宣传，提倡科学、文明、健康的生活方式。坚持重在建设的方针，开展好群众性精神文明创建活动。

　　加强社会主义民主与法制建设。进一步扩大基层民主，全面推行村务公开、厂务公开和政务公开，扩大公民有序的政治参与。广泛开展“四五”普法教育，增强全民法制观念和法律意识。加强执法队伍建设和管理，强化执法监督。正确处理新时期人民内部矛盾，做好人民来信来访工作，搞好矛盾纠纷排查，预防和妥善处理群体性事件。进一步强化社会治安综合治理，依法严厉打击各类犯罪活动和“法轮功”等非法邪教组织，扫除“黄、赌、毒”等社会丑恶现象。扎扎实实抓好安全生产，及时消除事故隐患，确保一方平安。

　　大力发展各项社会事业。加快科技创新体系建设，发展科技中介服务，完善创业孵化网络，加强知识产权保护，推动生产力促进事业发展，加速科技成果向现实生产力转化。积极发展高等教育和职业教育，大力支持山大威海分校和哈工大威海校区的发展，搞好威海职业学院、技术学院建设。抓好基础教育，推进素质教育，调整中小学布局，鼓励多种投入形式的社会办学，扩建改造市区部分中小学校，逐步解决市区中小学校舍紧张状况。加大文化设施投入，建设好千里海疆文化长廊，积极倡导高雅文化艺术，丰富群众文化生活。完善医疗卫生服务体系，强化卫生和药品监督，提高城乡居民的医疗保健水平。广泛开展全民健身活动，提高竞技体育水平。认真落实三大基本国策，加强人口、资源和环境保护工作。进一步做好新闻出版、广播电视、档案、史志、外事、侨务、民族、宗教、气象、减灾、妇女儿童、老龄及残疾人等项工作。加强国防教育，加大国防建设投入，搞好民兵预备役和“双拥”工作，增进军政、军民团结。

　　四、切实加强政府自身建设

　　各级政府及全体公务员，是现代化建设的组织者、推动者和实践者，重任在肩，使命神圣，一定要增强时代感、使命感和危机感，坚持勤政、廉政、优政，以与时俱进的创新精神，奋发有为的崭新姿态，昂扬向上的良好形象，带领全市人民在现代化建设的伟大事业中，打开新局面，创造新业绩。

　　紧跟时代，努力建设学习型创新型政府。全体政府公务员都要顺应时代潮流，把学习当成从政为民和工作创新的基本需要，努力学习和掌握马列主义、毛泽东思想、邓小平理论、“三个代表”重要思想和十六大精神实质，提高政治理论素质。努力学习和掌握与本职工作有关的方针政策、法律法规和其它相关知识，提高自身的业务素质和工作本领。要注意理论与实践相结合，切实把学与用、知与行统一起来，运用学习成果指导工作，研究问题，创新思路，创新方法，努力解决事关发展全局的现实问题。

　　转变职能，努力建设公共服务型政府。深入推进政府职能转变，使政府从过去的“以管理为主”转为“以服务为主”，真正把政府职能转到经济调节、市场监管、社会管理和公共服务上来。认真搞好第二轮行政审批制度改革，提高行政行为透明度，降低行政成本，提高行政效能。时刻牢记全心全意为人民服务的宗旨，完善为人民群众办实事制度，健全领导成员工作联系点制度，切实解决好事关人民群众切身利益的热点难点问题。

　　依法行政，努力建设民主法制型政府。加强依法行政，努力实现行政行为和程序的法定化，使政府从过去的“依权行政”转为“依法行政”，做全社会遵纪守法的表率。完善人民群众建议征集制度、重大事项社会公示制度和社会听证制度。各级政府要自觉接受同级人大及其常委会的监督，支持人民政协履行职能，主动加强与各民主党派、人民团体、无党派爱国人士的联系，充分发挥共青团、妇联、工会等组织的桥梁纽带作用，广泛听取各方面的意见和建议。

　　改进政风，努力建设勤政廉洁型政府。要坚持从严执政，认真落实廉洁自律规定，完善监督、约束、防范机制，继续治理纠正行业不正之风，加大从源头上消除腐败的力度。继续发扬党的优良传统，广泛开展“两个务必”教育，始终保持清醒头脑，增强忧患意识，自觉抵制腐朽思想的侵蚀和不正当利益的诱惑。坚持重实际、说实话、办实事、求实效，坚决反对形式主义、官僚主义、虚报浮夸等不良作风，深入基层和群众，加强调查研究，实行面对面的指导和服务。突出思想建设、效能建设和作风建设三个重要环节，继续深入开展“优政建设年”活动，以优政造福群众，以优政促进发展，以优政实践“三个代表”重要思想。对安排的工作，都要层层分解任务，明确目标和责任，加强效能监督检查，确保各项任务落到实处。

　　各位代表！肩负现代化建设的光荣使命，憧憬威海的美好未来，我们豪情满怀，信心百倍。让我们以党的十六大精神为指针，高举邓小平理论伟大旗帜，深入实践“三个代表”重要思想，紧密团结在以胡锦涛同志为总书记的党中央周围，在省委、省政府和威海市委的正确领导下，按照市十二届一次党代会的部署，团结和带领全市人民，解放思想，实事求是，与时俱进，开拓创新，为建设全面小康和现代化的新威海而努力奋斗！
